# Pulmonary Exacerbations in Pediatric Patients: Retrospective Study in a Portuguese Cystic Fibrosis Center

**DOI:** 10.3390/children9020157

**Published:** 2022-01-26

**Authors:** Rosa Cardoso, Ana Lúcia Cardoso, Telma Barbosa

**Affiliations:** 1 Instituto de Ciências Biomédicas Abel Salazar, Universidade do Porto, 4050-313 Porto, Portugal; 2 Cystic Fibrosis Reference Center, Centro Materno-Infantil do Norte, Centro Hospitalar Universitário do Porto, 4050-651 Porto, Portugal; ana.ld.cardoso@gmail.com (A.L.C.); telmab@gmail.com (T.B.)

**Keywords:** cystic fibrosis, pulmonary exacerbations, infection, lung function

## Abstract

(1) Background: Cystic fibrosis (CF) is a multisystemic disease caused by mutations in the cystic fibrosis transmembrane conductance regulator (CFTR) gene. Lung disease, the leading cause of morbimortality, is marked by acute worsening of symptoms—such as pulmonary exacerbations (PEx). The objectives of this study were: Identifying the frequency of PEx in pediatric CF patients; Characterizing each PEx; Finding association between the frequency and characteristics of the PEx and patients’ features. (2) Methods: Retrospective analysis of all PEx from a period of January 2015 to December 2019 in a group of pediatric patients from a single CF center. Data were collected from medical records. Descriptive statistics and chi-square/Fisher’s test were used. (3) Results: Thirty-four pediatric patients contributed to the total sample used in this study and 198 PEx were identified, median of 1.0 PEx/patient/year. Most frequent PEx symptoms were increased cough (93.9%) and change in secretions (88.4%), most common pathogens were *Staphylococcus aureus* (54.9%) and *Pseudomonas aeruginosa* (24.9%). The majority were treated as outpatient (85.9%). Most common antibiotics included amoxicillin/clavulanate (35.9%) and ciprofloxacin (22.7%). Outcome was favorable in all PEx. (4) Conclusion: Results were consistent with what has been described in literature. More studies are necessary for a better characterization of CF PEx, in order to develop standardized protocols for their management.

## 1. Introduction

Cystic fibrosis (CF) is a life-shortening multisystemic monogenetic disease caused by mutations in the cystic fibrosis transmembrane conductance regulator (CFTR) gene. It is the most common autosomal recessive cause of early mortality in Caucasians worldwide [1,2].

Among Caucasians, CF occurs in approximately 1 in 3000 to 4000 live births and it is estimated that 1 in 25 to 30 white persons are carriers of a pathogenic mutation of the CFTR gene [2]. In Portugal, the annual incidence is approximately 1 in 9000 live births and, in 2017, 341 patients were listed in the European Cystic Fibrosis Society Patients Registry (ECFSPR) [3,4].

The CFTR gene, located in chromosome 7, encodes for the CFTR protein, which functions as an ATP-gated chloride channel and as a bicarbonate channel [5]. It regulates the flow of ions across the apical surface of epithelial cells and it is widely distributed across several epithelial tissues [6]. More than 2000 potentially pathogenic mutations of the CFTR have been described [7]. The most common CF causing mutation is the f508del, a class II mutation that is present in up to 90% of people with CF [1,8]. 

The diagnosis of CF is based upon compatible phenotype or, more recently, newborn screening (NBS), both requiring biochemical or genetic confirmation. The Portuguese NBS program is a public health program that started in 1979, which encompasses 26 disorders, including inborn errors of metabolism, congenital hypothyroidism and, since 2013, CF. 

Clinical manifestations of CF include lung disease, pancreatic disease, liver disease, chronic pansinusitis, nasal polyposis, reduced fertility, reduced bone mineral density and increased salt content in sweat gland secretions [1,5]. Lung disease, which remains the leading cause of morbidity and mortality in patients with CF, begins in infancy [1]. Compromised ion transport results in dehydrated, thick secretions and, consequently, impaired mucociliary clearance, which leads to obstruction of the small airways, rendering the airways vulnerable to infection [9]. The continuous cycles of infection predispose to inflammation, scarring and architectural changes, making the lung even more prone to infection with different, some highly pathogenic, microorganisms, not usually present in healthy lungs. Over the past decades, considerable changes in the epidemiology of pathogens in CF patients have been described, possibly due to improved laboratory methods, molecular identification strategies, increased life expectancy and selective pressure from antimicrobial agents, among other factors [1,10]. Some of the most common pathogens include *Pseudomonas (P.) aeruginosa*, *Haemophilus (H.) influenzae*, *Staphylococcus (S.) aureus*, *Burkholderia (B.) cepacia*, *Stenotrophomonas (S.) maltophilia* and nontuberculous mycobacteria. *H. influenzae* is more common in infants and preschool-aged children, while *P. aeruginosa* becomes more frequent with age [1]. Chronic respiratory infection results in progressive loss of lung function and decreased survival, marked by acute worsening of symptoms, with cough, increased sputum production, dyspnea, malaise, anorexia and weight loss, called pulmonary exacerbations (PEx) [1].

Conventional long-term treatment aims to maintain optimal lung health and nutritional status [1]. For both pulmonary and gastrointestinal manifestations, treatment is lifelong and begins when the diagnosis is established. Regarding lung disease, treatment focuses mainly in controlling chronic airway infection and airway clearance of secretions. For the first aspect, routine cultures of respiratory secretions and targeted antimicrobial treatment are key. As for the latter, treatment is based in physical therapy techniques, (e.g., postural drainage and percussion, oscillating positive expiratory pressure, high frequency chest wall oscillation and exercise) and inhaled therapies to thin viscous mucus, such as recombinant human DNase (dornase alfa) and hypertonic saline [5]. Alongside conventional therapies, CFTR modulators (e.g., lumacaftor-ivacaftor) represent an important advance in management of CF, as instead of treating the consequences of the CFTR dysfunction, they target the production or function of the mutant CFTR protein, with the potential of dramatically changing the natural history of this disease [11,12].

Earlier diagnosis, especially with the advent of widely spread NBS programs, and therapeutic advances in CF have led to substantially improved survival rates. A child born today with CF can have a reasonable expectation to live into the sixth decade, with adult patients representing approximately half of the CF population [4]. As patients live longer, quality of life becomes an even more important factor to consider in the management of this condition [13]. One factor that dramatically influences the quality of life of these patients is the burden of treatments and long course intravenous antibiotics, often requiring hospital admission, most of them for PEx. PEx are extremely common and become more frequent with age and lung function decline [14].

Despite their importance and profound impact on the morbidity and quality of life of individuals with CF, although there are some studies focusing on trying to find a definition for PEx, a consensus definition of what constitutes a PEx has not yet been established. Furthermore, the approach to PEx diagnosis may be relatively consistent to a single clinician, but when it comes to different clinicians, the absence of a standardized approach may lead to substantial inconsistency [14].

The present study was conducted with the following objectives: Identifying the frequency of PEx in a group of pediatric CF patients; Characterizing each PEx, describing the clinical presentation, management and outcomes; Finding an association between the frequency and characteristics of the PEx and patients’ demographic and clinical features.

## 2. Materials and Methods

A retrospective observational study was conducted in a sample of pediatric patients from the CF center of a Northern Portugal University Hospital Center. Data on every PEx identified over a 5-year period (1 January 2015 to 31 December 2019) were collected. 

Sample selection was based on the following inclusion criteria: pediatric patients (age up to 17 years and 365 days old) with established CF diagnosis and follow up of at least 1 year in the CF Center during the study timeframe. Exclusion criteria included lung transplant, CFSPID/CRMS diagnosis and transition to adult care/transfer to other center before the beginning of data collection (2020). 

Similarly to what has been used in other studies, PEx identification was based on CF physician defined requirement to intervene with new antibiotic therapy, either oral, intravenous and/or inhaled, for at least one of the following signs and symptoms: increased cough, increased dyspnea, change in sputum volume or characteristics, new or increased hemoptysis, increased malaise, fatigue or lethargy, temperature over 38 °C, anorexia, weight loss, sinus pain or tenderness, change in sinus discharge, change in physical findings on chest examination and auscultation, reduced lung function and radiographic changes, as well as identification of a specific microorganism in sputum [15,16].

Data were collected from medical records of urgent and planned outpatient visits and hospital admissions. The retrieved information included demographic characteristics of the patients (age, gender, ethnicity and date of beginning of follow-up in the CF center), anthropometric data, disease characteristics (type of CFTR mutation, age and form of diagnosis, CF manifestations, comorbidities, state of persistent infection with specific microorganisms, long term treatment and lung function (the chosen parameter was Forced Expiratory Volume in one second percentage of predicted (FEV1% predicted)) and PEx characteristics (signs and symptoms, workup, treatment and outcome information). 

Age and anthropometric measurements selected for each year corresponded to the last outpatient visit and FEV1% predicted to the best measurement of each year. The state of persistent infection was defined as more than 50% positive sputum samples collected during the last 12 months or at least four positive sputum samples during the same period, as has been used in the European Cystic Fibrosis Society Patient Registry (ECFSPR) [4].

Each patient was attributed an identification code and three different databases were created, with different purposes:

- Sample characterization: database including demographic and disease characteristics from each patient, with one entry per patient. Patients’ clinical features that might have varied throughout the study timeframe, including lung function, presence of persistent airway infection with specific microorganisms and body mass index (BMI) z-score, were registered per year of study (2015–2019);
- PEx and hospital admission frequency determination: database with one entry per patient per year, with the patient identification code, year of study and number of PEx and hospital admissions for PEx in each year. Each entry included some relevant demographic and clinical patient information from the corresponding year, for comparison of the frequency of PEx and admissions for PEx between different groups;
- PEx characterization: database with one entry per PEx during the entire study timeframe, including patient identification code, signs and symptoms, workup and treatment information. For each entry, gender, patient age and FEV1% predicted at the time of the PEx were included, for comparison of the PEx characteristics between groups.

For statistical analysis, some variables were organized into groups, classified as: age as infants/children (under 10 years of age) and teenagers (10 years or older), genotype as f508del homozygous, f508del heterozygous and other genotypes, age of diagnosis as early diagnosis (first year of life) and later than the first year of life, lung function as FEV1% predicted below 80 and 80 or higher, nutritional status (using BMI z-scores) as underweight (BMI z-score less than −2.0) and normal/overweight (BMI z-score of −2 or higher). 

Finally, one PEx was randomly selected from each patient. These pulmonary exacerbation characteristics were registered in another database with the same variables as the PEx characterization database. This group of PEx was analyzed separately to understand if the features of this group of PEx were the same as the general PEx characteristics. 

Prior to its conduction, the study was approved by the hospital center Ethics Committee. Participation in the study was voluntary, and the parents and patients (when developmentally appropriate) were informed, in oral and written form, of the objectives of the study and all its procedures. Written consent was requested from each parent (for patients younger than 16 years old) or patient (if 16 years of age or older), with guarantee of absolute confidentiality of all collected data, which would later be analyzed anonymously. Patients between 12 and 16 years old signed a written assent form. Direct contact with patients was not needed for the study accomplishment, and the information was collected exclusively from clinical records. 

IBM^®^ Statistical Package for the Social Science Statistics^®^ version 26 was used for all statistical analysis. Descriptive statistical methods (frequencies and measures of central tendency and dispersion) were used for the description of patients’ demographic and clinical data and for the characterization of each PEx. The Shapiro–Wilk test was used to classify all the continuous variables according to distribution. The variables with Gaussian distribution were characterized using mean and standard deviation (SD) and the others with median and interquartile range (IQR). A comparative analysis was carried out between different groups, using Pearson chi-square and Fisher’s exact test (when Pearson chi-square was not applicable). In all statistical tests, a significance level of 0.05 was considered. 

## 3. Results

### 3.1. Sample Characterization 

The total sample constituted of 34 patients. For this analysis, database 1 was used, with 34 entries, each corresponding to a different patient. Follow up time during the study timeframe varied between 1 and 5 years, with 67.6% (*n* = 23) having been followed throughout the five years. Therefore, the number of patients included in each year of study varied between 23 (in 2015) and 34 (in 2019). The number of patients included in each year and the clinical features that might have varied throughout the study timeframe (lung function, presence of persistent airway infection and BMI z-score) are listed per year of study in Table 1. 

#### 3.1.1. Demographic 

The final sample was constituted by 52.9% female (*n* = 18). Mean age in 2015 and 2019 was 9.4 (SD = 3.3) and 11.7 (SD = 4.8), respectively. Caucasians were the most frequent ethnic group (91.2%, *n* = 31). Age throughout the study timeframe is described in Table 1. 

#### 3.1.2. Diagnosis and Genotype 

Age at diagnosis varied between 0 and 17 years (median zero, IQR 0–4), with seven patients diagnosed with the NBS program (20.6%). Eighteen patients (52.9%) were diagnosed in the first year of life.

The most frequent genotype was f508del homozygous (64.7%, *n* = 22), followed by f508del heterozygous (23.5%, *n* = 8) and other genotypes (11.8%, *n* = 4). 

#### 3.1.3. Clinical Features 

Mean FEV1% predicted was 93.2% (SD = 24.19) in 2015 and 82.3% (SD = 26.9) in 2019. The frequency of patients with FEV1% predicted below 80% in 2015 and 2019 was 31.6% (*n* = 6/19) and 32.1% (*n* = 9/28), respectively. 

Frequency of persistent infection with methicillin susceptible *S. aureus* (MSSA) in 2015 and 2019 was 31.9% (*n* = 9/23) and 69.7% (*n* = 23/34), respectively. No patients presented persistent methicillin resistant *S. aureus* (MRSA) infection. Persistent *P. aeruginosa* infection was present in three patients (13.0%) in 2015 and in four patients (12.1%) in 2019. No patients presented persistent *B. cepacia* infection. 

Mean BMI z-score at the beginning and end of the study was −0.38 (SD = 1.20) and −0.53 (SD = 1.18), respectively. The number of underweight patients was one (4.5%) in 2015 and four (12.5%) in 2019. 

FEV1% predicted, persistent airway infection frequency and BMI z-scores in each year are described in Table 1. 

Concerning CF related comorbidities, 70.6% of patients (*n* = 24) presented pancreatic insufficiency, three patients presented CF related hepatic disease without cirrhosis (8.8%) and no patients presented CF related diabetes, osteoporosis or osteopenia. Other comorbidities included asthma, which affected 61.8% of patients (*n* = 21), atopy, in 50.0% of patients (*n* = 17), triggers including pollens (*n* = 10) and dust mites (*n* = 10), and celiac disease, which affected two patients (5.8%). 

#### 3.1.4. Treatment 

Lumacaftor-ivacaftor was used in six patients (17.6%). All patients underwent regular chest physiotherapy and 38.2% (*n* = 13) used airway clearance devices (oscillating positive expiratory pressure and/or high frequency chest wall oscillation). All patients utilized nebulized hypertonic saline. DNase was used in 91.2% of patients (*n* = 31) and the same percentage used regular bronchodilator inhaled therapy. Half of the patients (50.0%, *n* = 17) were given inhaled antibiotics for more than three months at some point during the study timeframe and long-term azithromycin was used in two patients (5.9%). Inhaled corticosteroids were used in 61.8% of patients (*n* = 21). One patient was on home non-invasive ventilation and long-term oxygen therapy. The percentage of patients under pancreatic enzyme replacement was 70.6% (*n* = 24) and five patients (14.7%) used ursodeoxycholic acid. All patients were under fat-soluble vitamin supplements and DHA and five (14.7%) used nutritional supplements. Five patients (14.7%) had been proposed for lung transplant, from which four had a portacath insertion. 

### 3.2. Pulmonary Exacerbation Frequency 

For this analysis, database 2 was used, with 138 entries. Number of entries per patient varied between one and five. During the five years of study, a total of 198 PEx were identified. The total number of PEx per patient varied between one and 19 PEx (median 5, IQR 3–7), with a median frequency of PEx per year of 1.0 (IQR 0–2) and a median frequency of hospital admissions for PEx per year of 0.0 (IQR 0–0, range 0–3). 

Regarding the seasons of the year, 31.8% of PEx occurred in winter (*n* = 63), 31.8% in autumn (*n* = 63), 23.2% in spring (*n* = 46) and 13.1% in summer (*n* = 26). 

The percentage of years with three or more PEx and at least one hospital admission was compared between groups of gender, age, genotype, age at diagnosis, diagnosis through NBS, lung function, nutritional status and weight variation. 

#### Comparison between Groups 

Number of PEx per year in females was significantly higher than in males (*p* = 0.003, Table 2), as well as the frequency of hospital admissions (*p* = 0.001, Table 2). 

Patients with early diagnosis had more hospital admissions than those diagnosed later than the first year of life (*p* = 0.031, Table 2). The same tendency was found in the number of PEx per year, although not statistically significant. On the other hand, patients diagnosed through NBS, these patients revealed a tendency towards having less PEx and hospital admissions per year, although this comparison was not statistically significant. To clarify these results, the frequency of f508del homozygous patients was compared between the groups of patients diagnosed during and after the first year of life, not through NBS, and although not statistically significant, there was tendency for this frequency to be higher in the first group (81.8% vs. 62.5%). 

Regarding lung function, patients with a FEV1% predicted below 80% had more PEx per year (*p* = 0.003, Table 2), as well as hospital admissions (*p* < 0.001, Table 2) when compared to those with a higher FEV1% predicted. 

When comparing patients with persistent MSSA infection to patients without, there was a significant difference regarding the number of PEx per year (11.9% vs. 25.9%, *p* = 0.034, Table 2) and hospital admissions (7.1% vs. 24.1%, *p* = 0.005, Table 2). Considering persistent infection with *P. aeruginosa*, hospital admissions were more frequent in patients with this infection (41.7% vs. 11.1%, *p* = 0.003, Table 2) and there was a tendency for these patients to have more PEx per year (25.0% vs. 16.7%), although not statistically significant. 

Considering patients treated with lumacaftor/ivacaftor, this group had significantly more PEx per year (44.4% vs. 10.8% in patients not under this therapy, *p* < 0.001), as well as hospital admissions (37.0% vs. 8.1% in the other group, *p* < 0.001). 

Underweight patients had more hospital admissions than normal/overweight patients (*p* = 0.050, Table 2), although those whose BMI z-score increased from one year to the other had more PEx per year (*p* = 0.006, Table 2). 

Lastly, there was a significant difference between patients who were proposed for lung transplant and those who were not, with the first group having more PEx (*p* < 0.001) and hospital admissions (*p* < 0.001) than the second. 

All results of comparisons made are listed in Table 2.

### 3.3. Pulmonary Exacerbation Characterization 

#### 3.3.1. Signs and Symptoms 

The most frequently described symptoms were increased cough (93.9%, *n* = 186) and increase/change in secretions (88.4%, *n* = 175). The least frequent ones were hemoptysis (15.7%, *n* = 31) and chest pain (4.5%, *n* = 9). Fever was present in 25.3% of the PEx (*n* = 50). Loss of weight was identified in 28.3% of cases (*n* = 56), low SpO_2_ (SpO_2_ < 95%) in 7.1% (*n* = 14/185), tachypnoea and/or chest retractions in 32.3% (*n* = 64) and changes in basal pulmonary auscultation in 78.8% (*n* = 156). Frequency of all symptoms and signs are described in Table 3.

When compared by age, hemoptysis was more frequent in teenagers (26.0% vs. 4.3% in children and infants, *p* < 0.001), as well as weight loss (34.6% vs. 21.3%, *p* = 0.037). Other comparisons were made, although not statistically significant. 

For lung function, patients with a FEV1% predicted below 80% were more symptomatic that those with a higher FEV1% predicted, having had more increase or change in secretions (96.2% vs. 82.8%, *p* = 0.005), dyspnea/exercise intolerance (40.5% vs. 16.1%, *p* < 0.001), loss of weight (39.2% vs. 23.0%, *p* = 0.023), hypoxemia (10.6% vs. 1.1%, *p* = 0.021) and chest retractions or tachypnea (58.2% vs. 6.9%, *p* < 0.001). 

#### 3.3.2. Workup 

The most frequently requested examination was respiratory tract specimen collection for culture, in 87.4% of cases (*n* = 173). The most common form of collection was spontaneous sputum and sputum induction with hypertonic saline was used when necessary. MSSA was the most frequently identified agent (54.9%, *n* = 95/173), followed by *P. aeruginosa* (24.9%, *n* = 43/173), *S. maltophilia* (14.5%, *n* = 25/173), *H. influenzae* (11.0%, *n* = 9/173) and *A. xylosoxidans* (8.1%, *n* = 14/173). *Aspergillus* spp. was detected in 12.7% of cases (*n* = 22/173) and *Candida* spp. in 4.6% (*n* = 8/173). When compared by age, *P. aeruginosa* was more frequently detected in teenagers than in infants and children (32.6% vs. 16%, *p* = 0.012). The same happened with *S. maltophilia* (19.6% in teenagers vs. 8.6% in infants and children, *p* = 0.041). *Moraxella catarrhalis*, on the other hand, was detected in 7.4% of PEx in infants and children (*n* = 6/81) and not detected in teenagers (*p* = 0.008). Regarding gender differences, *S. maltophilia* and *P. mirabilis* were more frequently identified in females and the opposite was verified with MSSA. Other comparisons were made, although not statistically significant.

Viral PCR in the nasopharyngeal wash was performed in 11.1% of cases (*n* = 22). The most frequently identified agents were Rhinovirus (27.2%, *n* = 6/22) and Respiratory syncytial virus (22.7%, *n* = 5/22). 

Chest X-ray was requested in 35.4% of PEx (*n* = 70), from which 60.0% (*n* = 42/70) displayed changes from the previous exam. Computed Tomography (CT) thoracic angiogram was requested in one case. 

Within patients who were admitted for inpatient treatment (14.1%, *n* = 28), blood culture was requested in 50.0% of cases (*n* = 14/28), from which none was positive. C-reactive protein (CRP) was requested in 81.5% of cases (*n* = 23/28), and was higher than 50.0 mg/L in seven cases (30.0%). A white blood cell count was requested in 89.3% of cases (*n* = 25/28), from which nine (36.0%) presented leucocytosis (leukocytes count higher than 15.000/μL). 

#### 3.3.3. Treatment 

Patients were admitted for inpatient treatment in 14.1% of PEx (*n* = 28). A notable difference was found between the inpatient and outpatient treatment groups in the frequency of patients with FEV1% predicted below 80%, significantly higher in the inpatient treatment group (81.0% vs. 42.8%, *p* = 0.001). There was also a significantly higher percentage of females within the inpatient treatment group than males (85.7% vs. 14.3%, *p* = 0.001). Furthermore, patients who were admitted for inpatient treatment had dyspnea/exercise intolerance and presented loss of weight, chest retractions/tachypnoea and hypoxemia more frequently than those in which an outpatient approach was preferred. Regarding respiratory specimen culture results, *P. aeruginosa* and *S. maltophilia* were more frequently detected in patients admitted into hospital. 

Oral antibiotics were the first choice in 90.4% of PEx (*n* = 176), and 5.6% (*n* = 11) required a switch to intravenous treatment. Intravenous antibiotics were used in 14.6% of PEx (*n* = 29) and were the first choice in 62.0% of these cases (*n* = 18/29). Intravenous antibiotics were administered at home in two cases, with support from the hospital home health care team. Inhaled antibiotics were used in 35.3% of cases (*n* = 70) and were used exclusively in one case (0.5%). 

The most frequently used antibiotics were amoxicillin/clavulanate (35.9%, *n* = 71), ciprofloxacin (22.7%, *n* = 45), flucloxacillin (17.7%, *n* = 35) and cotrimoxazole (10.1%, *n* = 20). In 17.7% of cases (*n* = 35), more than one antibiotic was prescribed. Number of prescribed antibiotics per PEx varied between one (82.3%, *n* = 163) and four (1.0%, *n* = 2). 

Allergic bronchopulmonary aspergillosis was diagnosed in five cases (2.5%), which were treated using antifungal therapy and systemic corticosteroids. 

Chest physiotherapy and caloric intake were intensified in all cases. 

#### 3.3.4. Outcome 

Outcome was favorable in all PEx. In a minority of cases (13.1%, *n* = 26), antibiotic treatment delivery was changed for adjustment to a different isolation in the sputum culture (when compared to previous cultures) and in 5.6% (*n* = 11) the treatment route was changed from oral to IV for mild improvement/worsening of the symptoms. One episode demanded admission to the Pediatric Intermediate Care Unit, post chest CT guided arterial embolization for hemoptysis control, with a length of stay of 2 days and a favorable outcome. No patients required mechanical ventilation or other measures of advanced life support. 

#### 3.3.5. Randomly Selected Pulmonary Exacerbation Group Analysis

When analyzing a group including one randomly selected PEx from each patient, no relevant differences were found between this group and the entire PEx sample.

## 4. Discussion

### 4.1. Context of the Study and Sample 

Although PEx have become increasingly recognized as an important aspect of CF lung disease, with their negative impact in the outcomes of CF patients being well established, there is a gap in literature when it comes to characterizing these episodes and defining their optimal approach. 

To the best of our knowledge, there are no published studies analyzing the characteristics and frequency of PEx in the Portuguese pediatric CF population. Furthermore, most internationally published studies focused mainly on characterizing the outcomes of PEx, and not their characteristics and diagnostic approach, and used definitions of PEx that include treatment with IV antibiotics as a criterion, failing to characterize milder PEx, treated in the outpatient setting, which seem to be the most frequent in pediatric age. 

We found two American studies with similar objectives to our own, one by Cogen et al. [17], a multicenter retrospective study including approximately five thousand patients, focusing on characterizing the consistency and variability in the inpatient management of CF-related PEx (excluding the outpatient setting), and one by Hoppe et al. [16], a prospective study aiming to characterize the frequency and clinical impact of PEx in infants and preschool aged children with CF (excluding older children and teenagers). 

Although the current study was of smaller sample compared to other studies, namely the one by Cogen et al. [17], the main strengths of our study rely on the fact that it aims to fill an investigational gap and that it uses a sample that, although small, represents 18.4% of the 185 Portuguese pediatric CF patients registered in the ECFSPR 2017 Annual Report and includes all eligible patients from one of the two CF centers in the north of the country [4].

A comparison between the characteristics of our sample with what was described in the ECFSPR 2017 report can provide a framework to understanding our results. Similar or only slightly different characteristics were found for the following variables (ECFSPR report vs. our sample): distribution among genotypes (percentage of f508del homozygous approximately 50.0% vs. 64.7%), mean FEV1 (83.0% in 6–17 year-old patients vs. 93.2%/82.3% in 2015/2019), frequency of patients with FEV1% predicted below 80% (approximately 35.0% in 6–17 year-old patients vs. 31.6/32.1% in 2015/2019), frequency of pancreatic insufficiency (82.0% vs. 70.6%) and mean BMI z-score (−0.40 vs. −0.38/−0.53 in 2015/2019). The main differences found include the frequency of persistent infection with *P. aeruginosa* (21.7% of the pediatric patients vs. 13.0/12.1% in 2015/2019), MSSA (46.2% vs. 39.1/69.7% in 2015/2019) and *B. cepacia* (6.0% of the pediatric patients vs. none) [4]. These differences are worth noting, as they might have influenced some decisions in the PEx management, such as antibiotic selection and hospital admission rate. It is also worth hypothesizing on what might have been behind these differences. One hypothesis to justify the lower *P. aeruginosa* and *B. cepacia* chronic infection rates may be the potential differences among CF centers in the policies of crossed infection prevention in CF patients. On the other hand, the higher frequency of MSSA chronic infection in our center might be related to two main factors. Firstly, contrarily to some centers, MSSA prophylaxis with flucloxacillin is not a common practice in our center. This practice is highly controversial, as it may be associated with the emergence of antibiotic resistance, with higher rates of MRSA chronic infection (inexistent in our center), not having, at the same time, clear benefits. Furthermore, the deleterious role of MSSA chronic infection in pulmonary function, contrarily to what happens with MRSA and *P. aeruginosa* chronic infection, is not completely clear [18]. Secondly, co-infection with *P. aeruginosa* and MSSA is very complex, with some studies showing that within the CF lung, competitive strategies may lead to changes in the metabolism of MSSA, making it less able to survive in the context of *P. aeruginosa* chronic infection [19]; therefore, the lower rates of *P. aeruginosa* chronic infection may have partially contributed to the higher MSSA chronic infection rates verified. 

### 4.2. Pulmonary Exacerbation Frequency 

Our study found a median frequency of PEx of one per year and a median inpatient PEx of zero per year. These frequencies are similar to what was found in the two American studies, with Cogen et al. reporting a mean number of inpatient PEx per patient of 3.8 over a period of 5 years, corresponding to a mean frequency of 0.8 per year, and Hoppe et al. reporting a median number of two PEx over a period of 2 years, corresponding to one PEx per year [16].

We found a predominance of PEx in autumn/winter months, followed by spring, with fewer PEx in summer. Although this was not explored in the two aforementioned studies, this result can be supported by the fact that respiratory viral infections (more frequent in the autumn/winter months) and atopy (with a considerate number of patients in our sample being sensitized to pollens) have been described as triggers to PEx in CF [20,21].

The difference between males and females was also a relevant finding, with the latter ones having a higher frequency of PEx and hospital admissions. This difference between genders, with a tendency of the females to a more severe course of disease, has been described in literature. Even though no studies have been published analyzing gender difference in children, according to a study by Harness-Brumley published in 2014, women with CF have worse outcomes than men, and are more predisposed to infection by respiratory pathogens, starting in younger ages, which leads to a lower life-expectancy. This may be related to differences in immunologic response to infection, estrogen influence in pathogen resistance mechanisms and the effect of sex hormones in the epithelial secretion of chloride and sodium in the respiratory tract [22]. Hoppe’s study found no differences in PEx frequency between males and females [16]. This may be related to the fact that the study included a younger sample, with shorter course of disease and possibly less chances for the gender differences to become evident.

Patients with a FEV1% predicted lower than 80% were found to have more PEx per year, as well as hospital admissions. This was one of the objectives of Hoppe’s study, although no association between PEx frequency and lung function was found [16]. This may be due to a higher number of older patients included in our study, who have lived with CF for longer and had a more intense lung function decline, having a lower FEV1% predicted. 

Our results revealed a higher frequency of hospital admissions in patients who were diagnosed in the first year of life, and a tendency for these patients to have more PEx per year. However, the same tendency was not perceived in patients who were diagnosed through NBS. This may be due to the fact that patients diagnosed in the first year of life and not through NBS are diagnosed on the basis of symptom presentation (e.g., meconium ileus) which means they have earlier manifestations of CF and therefore might have more severe forms of the disease, leading to worse outcomes. This hypothesis is supported by the fact that the frequency of f508del homozygous patients (typically associated with a worse outcome when compared to other genotypes) was higher in the group of patients diagnosed during the first year of life, when compared to patients diagnosed later than that [1]. On the other hand, patients diagnosed through NBS have earlier access to routine CF care, even before having clinical manifestations, which leads to a better control of the disease. 

Another interesting finding was the difference between patients with persistent infection with MSSA and those without, with the second group having a higher number of PEx and hospital admissions per year. This result goes accordingly to the fact that MSSA infection, in the absence of concomitant pathogens, has been described as a marker for milder disease [16]. The opposite was verified in persistent *P. aeruginosa* infection, with patients having more PEx and hospital admissions per year. This result was expected, as infection with *P. aeruginosa* has been clearly related to increased morbidity and mortality in CF patients, with recurrent PEx and a gradual decrease in lung function. 

Patients proposed for lung transplant also had more PEx and hospital admissions per year, which goes accordingly to the fact that these patients are usually more severely affected, with significantly compromised lung function. 

Patients treated with lumacaftor/ivacaftor had significantly more PEx and hospital admissions per year when compared to other patients. This was probably due to a selection bias, as the use of this drug in our community is very recent and was started through an early access program, reserved for more severely affected patients, with significant deterioration of lung function. Although the benefits of this drug were clear on an individual basis, with patients having an improvement in nutritional status and a decrease in lung function decline and frequency of PEx, consistently with what has been described in literature [23], it would be worth repeating this analysis in the future, when CFTR modulators are accessible to a larger number of patients, at an earlier age/stage of the disease, before structural lung disease, chronic infection and lung function decrease have set in, similarly to what already happens in some countries [24].

Finally, underweight patients had more hospital admissions than normal/overweight ones. This association has been described in literature, with poor growth and malnutrition, contributing significantly to increased morbidity in these patients [25]. On the other hand, patients who had an increase in weight had more PEx per year. The reason behind this may be the fact that patients who have more PEx require more hospital visits, allowing closer monitoring of nutritional status and adhesion to treatment, namely pancreatic enzymes and supplements, even though no similar results were found in literature, including in Hoppe’s study [16], where no association was found between BMI and the frequency of PEx. 

### 4.3. Pulmonary Exacerbation Characterization 

As mentioned previously, there is a scarcity of recent studies characterizing the symptoms and signs presented in PEx in the pediatric population. The most frequent symptoms and signs found in our study corresponded to the clinical features usually associated with PEx stated in several international guidelines, namely the CF Foundation Clinical Care Guidelines and the Royal Brompton Hospital Clinical Guidelines: Care of Children with Cystic Fibrosis [24,26]. It is worth emphasizing the differences found between children and teenagers, with hemoptysis and loss of weight significantly more frequent in the latter ones, and between patients with different degrees of lung function deterioration, with dyspnea, chest retractions and/or tachypnoea, loss of weight and hypoxemia being more common in patients with a FEV1% predicted inferior to 80%. This reinforces the importance of characterizing PEx taking age and disease characteristics into account. 

Similarly to what was described in previous studies [16,17], the most frequent pathogens found in respiratory specimen cultures included MSSA, *P. aeruginosa*, *S. maltophilia*, *H. influenzae* and *Aspergillus* spp. Least commonly described pathogens were also found, such as *Cupriavidus pauculus*, *Sphingomonas paucimobilis*, *Acinetobacter jonsonii* and *Rhizobia radiobacter*, highlighting the fact that CF clinicians must be continuously adapting to the changes in epidemiology of respiratory bacteriology and the emergence of certain agents and resistant species. The fact that increasing age makes infection with some specific agents more likely was also documented in our results, with *P. aeruginosa*, *S. maltophilia* and *A. xylosidans* more frequently identified in teenagers [1]. Regarding differences between genders, MSSA was more prevalent in males and *S. maltophilia* and *P. mirabilis* was more frequent in females. This may be due to the fact that females tend to acquire infections at an earlier age than males, becoming infected by some agents earlier than males [22].

An outpatient approach was preferred in the vast majority of cases (85.9%). This was similar to what was described in Hoppe’s study, with 85% of PEx treated in the outpatient setting. According to literature, the choice for an outpatient treatment, when clinically appropriate, has many potential benefits, not only in reducing nosocomial infections or negative outcomes, but also in patient and family satisfaction [10]. In our study, an inpatient approach was preferred in patients with lower FEV1 and some specific signs and symptoms, such as dyspnea, weight loss, hemoptysis, hypoxemia and chest retractions. This approach may have some advantages, such as a wider range of treatment possibilities, as some antibiotics are only available for IV administration, and a significantly greater lung function recovery, as has been described in literature [27], making it particularly suitable for patients with low baseline FEV1. 

With respect to antibiotic therapy, CF patients have some peculiarities, namely drug metabolism, with higher doses often necessary, being treatment outlining usually more complex than for pulmonary infections in other clinical contexts. Treatment for CF PEx is also more complex than other pulmonary infections. For instance, antibiotics selection is based not only on pathogens prevalence (in CF and general community), but also on patient previous/current cultural exams. Selecting what worked best for the last PEx in each patient is also recommended [9]. The most frequently used antibiotics included penicillin, fluoroquinolones and cotrimoxazole, which was consistent with the results of Hopper’s study. In most cases, a single antibiotic agent was prescribed, while multiple antibiotics were used in 17.7% of cases. Although, traditionally, CF guidelines have recommended the use of multiple antibiotics for the treatment of PEx (still a common practice for the inpatient setting), there is poor evidence that this practice leads to a greater improvement in lung function when compared those treated the use of a single drug, especially in younger patients [1].

### 4.4. Limitations of the Study 

Given the fact that it was a retrospective study, there were some limitations inherent to its methodology, such as the inability to apply standardized data collection forms and to use published criteria/scores for the diagnosis of PEx, such as the Fuchs criteria or the Rosenfeld score [14,28].

As most studies on this topic focus mainly in PEx outcomes, a frequently analyzed parameter is the impact of each PEx in lung function. This information was not explored in our study because, although lung function testing was performed in some of the included patients in context of a PEx, it was not assessed in a systematized way, thus not allowing a systematized analysis of this factor. This could be either due to characteristics of the PEx, such as the presence of hemoptysis, in which performing spirometry in the acute setting could predispose to a new episode of bleeding, patients’ age (and therefore inability to collaborate with the exam). Nevertheless, it is worth mentioning that there are no consistent recommendations considering frequency of lung function testing in PEx or a standardized way of incorporation of spirometry results into decision making [17]. The frequency of lung function testing was, in fact, the parameter for which Cogen et al. found more variability among centers [17]. Furthermore, spirometry is less sensitive in evaluating structural damage in milder lung disease, particularly in young children, and, consequently, abnormalities might only become apparent after longer follow up times [16]. 

As for sample size, although representing nearly 20% of the country’s pediatric CF patients, it is not representative of the entire population and the results cannot be generalized. 

### 4.5. Perspectives for the Future 

The entity we call PEx is a lot more heterogeneous than is commonly acknowledged. There has been important progress over the years in understanding PEx, although unexplored territory is still immense and there are many gaps to fill. 

Performing this type of study in a larger scale, using wider samples, more representative of the CF population, would be relevant not only for the development of a universal definition of PEx, but also for providing information for the conception of standardized protocols for their diagnosis and management, hence improving the quality of patient care and minimizing CF-related outcomes. 

Finally, due to the increase in life expectancy of patients with CF, especially with the emergence of new treatments, such as the CFTR modulators, disease characteristics may be changing and there may be a lot to discover as these patients grow older and live longer, which makes it crucial that investigation in this area continues. 

## 5. Conclusions

Despite the fact that few studies dedicated to the characterization of PEx have been published, making it difficult to find literature to compare our results with, most of them go accordingly to what has been described, such as the frequency of PEx and the epidemiology of respiratory pathogens. 

From the obtained results, one finding that stands out is the significant difference between genders, with females having more PEx, hospital admissions and being more frequently proposed for lung transplant. It has also been notable that patients with a lower FEV1% predicted have more PEx and hospital admissions and presented more severe symptoms. 

Furthermore, the differences in the presentation of PEx and respiratory pathogens identified between younger children and teenagers are also worth mentioning, as this emphasizes the need to expand our knowledge and investigation of PEx in the pediatric population, taking age and patient characteristics into account. 

## Figures and Tables

**Table 1:** Sample characteristics per year of study.

Variable	Year of Study				
	2015 (N = 23)	2016 (N = 26)	2017 (N = 27)	2018 (N = 29)	2019 (N = 34)
Age ^1^	Mean | SD | Range	Mean | SD | Range	Mean | SD | Range	Mean | SD | Range	Mean | SD | Range
	9.4 | 3.3 | 1–14	9.6 | 3.89 | 1–15	10.2 | 4.3 | 1–16	11.1 | 4.3 | 1–17	11.7 | 4.8 | 1–18
Children	Frequency: % (*n*)	Frequency: % (*n*)	Frequency: % (*n*)	Frequency: % (*n*)	Frequency: % (*n*)
	56.5 (13)	42.3 (11)	29.6 (8)	27.6 (8)	32.4 (11)
Teenagers	Frequency: % (*n*)	Frequency: % (*n*)	Frequency: % (*n*)	Frequency: % (*n*)	Frequency: % (*n*)
	43.5 (10)	57.7 (15)	70.4 (19)	72.4 (21)	67.6 (23)
FEV1% predicted	Mean | SD | Range | *n*	Mean | SD | Range | *n*	Mean | SD | Range | *n*	Mean | SD | Range | *n*	Mean | SD | Range | *n*
	93.2 | 24.19 | 46–126 | 19	85.3 | 25.3 | 43–119 | 20	85.1| 27.3 | 34–137 | 24	82.3 | 27.2 | 38–123 | 25	82.3 | 26.9 | 28–114 | 28
<80%	Frequency: % (*n*)	Frequency: % (*n*)	Frequency: % (*n*)	Frequency: % (*n*)	Frequency: % (*n*)
	31.6 (6)	40 (8)	29.2 (7)	40.0 (10)	32.1 (9)
Persistent infection	Frequency: % (*n*)	Frequency: % (*n*)	Frequency: % (*n*)	Frequency: % (*n*)	Frequency: % (*n*)
*S. aureus* ^2^	39.1 (9)	46.2 (12)	70.4 (19)	72.4 (21)	69.7 (23)
*P. aeruginosa*	13.0 (3)	3.8 (1)	3.7 (1)	10.3 (3)	12.1 (4)
*B. cepacia*	0 (0)	0 (0)	0 (0)	0 (0)	0 (0)
BMI z-score	Mean | SD | Range | *n*	Mean | SD | Range | *n*	Mean | SD | Range | *n*	Mean | SD | Range | *n*	Mean | SD | Range | *n*
	−0.38 | 1.20 −3.19–1.89 | 22	−0.47 | 1.18 −2.85–1.80 | 25	−0.47 | 1.13 −2.42–1.73 | 25	−0.44 | 1.05 −2.70–1.71 | 28	−0.53 | 1.18 −3.27–1.47 | 32
Underweight (BMI z-score <−2)	Frequency: % (*n*)	Frequency: % (*n*)	Frequency: % (*n*)	Frequency: % (*n*)	Frequency: % (*n*)
	4.5 (1/22)	16.0 (4/25)	12 (3/25)	7.1 (2/28)	12.5 (4/32)

^1^ By the end of each year; ^2^ only methicillin susceptible *Staphylococcus aureus* persistent infection was found.

**Table 2:** Comparison of frequency of PEx and hospital admissions between patient groups.

Patient Characteristics	Frequency: % (*n*) (N = 138)			
	≥3 PEx/Year (In at Least 1 Year)	*p*-Value	≥1 Inpatient Admission/Year (In at Least 1 Year)	*p*-Value
Sex				
Female	27.3 (18/66)	0.003 ^1^	24.2 (16/66)	0.001 ^1^
Male	8.3 (6/72)		4.2 (3/72)	
Age				
Children and infants	11.3 (7/62)	0.088 ^1^	9.7 (6/62)	0.208 ^1^
Teenagers	22.4 (17/76)		17.1 (13/76)	
Genotype				
f508del homozygote	20.8 (20/96)	N.A.	15.6 (15/96)	N.A.
f508del heterozygote	4.5 (1/22)		13.6 (3/22)	
Other	15.0 (3/20)		5.0 (1/20)	
Age of diagnosis				
Early diagnosis (1st YOL)	20.8 (15/72)	0.215 ^1^	20.8 (15/72)	0.031 ^1^
Later than 1st YOL	12.5 (7/56)		7.1 (4/56)	
Diagnosis through Newborn Screening				
Yes	9.5 (2/21)	0.531 ^2^	9.5 (2/21)	0.738 ^2^
No	18.8 (22/117)		14.5 (17/117)	
Lung function				
FEV1% predicted ≥80%	10.5 (8/76)	0.003 ^1^	3.9 (3/76)	<0.001 ^1^
FEV1% predicted <80%	32.5 (13/40)		30.0 (12/40)	
Lung transplant proposal				
Yes	47.6 (10/21)	<0.001 ^2^	57.1 (12/21)	<0.001 ^2^
No	12.0 (14/117)		6.0 (7/117)	
Nutritional status (N = 132)				
Normal/overweight (BMI z-score ≥ −2)	19.8 (21/106)	0.407 ^2^	10.4 (11/106)	0.050 ^2^
Underweight (BMI z-score < −2)	11.5 (3/26)		26.9 (7/26)	
Weight variation (N = 131)				
BMI z-score decrease	9.5 (7/74)	0.006 ^1^	14.9 (11/74)	0.894 ^1^
BMI z-score increase	28.1 (16/57)		14.0 (8/57)	
Persistent *S. aureus* infection				
Yes	11.9 (10/84)	0.034 ^1^	7.1 (6/84)	0.005 ^1^
No	25.9 (14/54)		24.1 (13/54)	
Persistent *P. aeruginosa* infection				
Yes	25.0 (3/12)	0.438 ^2^	41.7 (5/12)	0.003 ^1^
No	16.7 (21/126)		11.1 (14/126)	

^1^ Pearson Chi-square test; ^2^ Fisher’s exact test; N.A.—Not applicable.

**Table 3:** Symptoms and Signs.

Symptoms and Signs	Frequency: % (*n*) (N = 198)
Clinical History	
Increased cough	93.3 (186)
Increase/Change in secretions	88.4 (175)
Dyspnea/Exercise intolerance	29.3 (58)
Hemoptysis	15.7 (31)
Chest pain	4.5 (9)
Physical Examination	
Loss of weight	28.3 (56)
Fever	23.5 (50)
Hypoxemia ^1^	7.1 (14/185)
SpO_2_ 90–94%	5.6 (11)
SpO_2_ 85–89%	1.5 (3)
SpO_2_ < 85%	0 (0)
Chest retractions and/or tachypnoea	32.3 (64)
Basal	15.1 (30)
De novo	17.1 (34)
Changes in pulmonary auscultation	78.8 (156)

^1^ Excluded the PEx of a patient with long term oxygen therapy.
